# Statistical overview of the *Sniffin’ sticks* olfactory test from the perspectives of *anosmia* and *hyposmia*

**DOI:** 10.1038/s41598-025-93380-z

**Published:** 2025-03-15

**Authors:** László Sipos, Zsófia Galambosi, Sándor Bozóki, Zsombor Szádoczki

**Affiliations:** 1 Department of Postharvest, Commercial and Sensory Science, Institute of Food Science and Technology, Hungarian University of Agriculture and Life Sciences, 1118 Villányi út 29−43, Budapest, Hungary; 2 Institute of Economics, HUN-REN Centre of Economic and Regional Studies, 1097 Tóth Kálmán utca 4., Budapest, Hungary; 3 Research Laboratory on Engineering & Management Intelligence, HUN-REN Institute for Computer Science and Control (SZTAKI), 1111 Kende u. 13−17, Budapest, Hungary; 4 Department of Operations Research and Actuarial Sciences, Corvinus University of Budapest, 1093 Fővám tér 8, Budapest, Hungary

**Keywords:** Olfactory test, *Sniffin’ sticks*, TDI threshold, Identification, Number of alternatives, Balanced, Simulation, Diseases, Diagnosis

## Abstract

The *Sniffin’ Sticks* test is evaluated by summing the scores of threshold, discrimination, and identification subtests to establish an olfactory diagnosis (*anosmia*, *hyposmia*, *normosmia*). However, variations in thresholds, ranges and inconsistencies have been observed. Statistical analyses (distributions, quantiles), protocol simulation, and implementations of the *Sniffin’ Sticks* test were conducted. This study contributes a statistical revision: determining a cut-off point for the total TDI score, optimising the number of alternatives in the identification subtest, and equalising correct responses (frequency, balanced in time, subsequent pairs). The upper score threshold used to diagnose *anosmia* is lower than the typical first-order error thresholds applied in comparable assessments (*anosmia* ≤ 16.75 points, *hyposmia* ≥ 17 points at 90% confidence level). In the culturally adapted 16-pen *Sniffin’ Sticks* identification tests, the frequency of correct responses was aligned with that of the original test. However, the equalisation of the frequency of adjacent correct response pairs and the temporal occurrence of correct responses was not met in any test. The order of all correct answers that simultaneously satisfied all three matching conditions was determined. The practical significance of the results is that the recommended protocol of the *Sniffin’ Sticks* test system can be implemented in clinical practice with minimal modification.

**Supplementary Information:**

The online version contains supplementary material available at 10.1038/s41598-025-93380-z.

## Introduction

The *Sniffin’ Sticks* test system (Burghart Messtechnik GmbH, Holm, Germany), a clinically validated and widely referenced tool in the clinical literature, assesses olfactory function through three mandatory-choice subtests: (1) a subtest measuring the perception of odours at low concentrations (olfactory threshold (T)), (2) a subtest measuring the non-verbal discrimination of different odours (odour discrimination (D)) and (3) a subtest measuring the ability to name or associate odours (odour identification (I)). According to the protocol, three-minute breaks are included between the olfactory threshold, odour discrimination, and odour identification tests. The olfactory threshold and discrimination test is performed with 16–16 so-called pen triplets, while the identification test involves recognising 16 pen odours^1^.

The olfactory threshold is determined using a staircase technique. The assessor is presented with 16 different concentrations of the target odour: n-butanol (strong characteristic and mildly alcoholic odour) or 2-phenylethyl alcohol (rose-like odour). The assessor’s task is to select the pen containing the target odour from a set of three pens in a three-alternative forced choice (3-AFC) task. The test begins by presenting the pen with the highest concentration to familiarise the assessor with the odour. Subsequently, the test proceeds with the pen containing the weakest concentration. After an incorrect response, the concentration is increased by 2 olfactory thresholds until the odour is correctly identified twice in succession at the same concentration. The first turning point is the assessor’s first two correct responses, the second turning point is the first incorrect response for the weaker concentration of odours, and the third turning point is two correct responses for the higher concentration of odours. This process continues in this way until the seventh turning point is reached. In the standard version of the *Sniffin’ Sticks*, the olfactory threshold is calculated as the average of the dilution values obtained from the last four turning points^1–3^.

The discrimination test also uses pen triplets, with all pens containing odours at concentrations above the olfactory threshold. This test involves presenting two pens with the same odour and one with a different odour. The assessor’s task is to identify which odour differs from the other two. A correct answer is recorded if the assessor accurately selects the pen with the different odour^1^.

In the identification test, the assessor is asked to identify the presented odour by selecting one of four options provided on a card. The test can be administered through the right nostril, the left nostril, or both nostrils simultaneously. If testing is performed on only one nostril, the order of the pens should be changed. The not-tested nostril should remain covered during this process, ensuring that the tested nostril is not deformed. The total test score is calculated as the sum of correct answers from both subtests^1^.

The *Sniffin’ Sticks* tests are scored by summing the three scores (TDI score = T + D + I scores) on the subtests to determine the diagnosis. Different tests often use different cut-offs (TDI ≤ x) to make diagnoses: *anosmia* x ∈ [15, 16.5], functional *anosmia* x ∈ [15, 16.5], *hyposmia* x ∈ (30, 34.5), *normosmia* (30, 34.5) ≤ TDI. Every test provides a threshold for *anosmia* or functional *anosmia*, but not for both. *Anosmia* is defined as the inability to smell. In the case of specific *anosmia*, the individual is unable to smell a specific odour, whereas the vast majority of odours are normally perceived. Functional *anosmia* refers to a significantly reduced ability to smell, although some olfactory sensations may be present. However, this preception does not provide patients with a normal sense of smell that is meaningful in daily life^4^. Generally, the importance of these senses is only recognised when they are lost. It is estimated that about 5% of the population experiences functional *anosmia*. Individuals with a complete loss of olfaction (*anosmia*) respond entirely randomly to the olfactory tests. Patients with functional *anosmia* do not perceive an odour correctly. They perceive some olfactory information, but not enough to correctly identify the odour. The *Sniffin’ Sticks* diagnostic test can be used to categorise an individual’s olfactory perception (*anosmia*,* hyposmia*,* normosmia*) based on their total TDI score. The *Sniffin’ Sticks* diagnostic test is not suitable for exactly distinguishing between *anosmia* and the less clinically significant functional *anosmia*. Nonetheless, functional *anosmia* patients typically score higher on average than randomly responding *anosmia* patients^5–8^. Functional *anosmia* would reasonably be associated to higher threshold than that of *anosmia*. However, this is not reflected in the proposed ranges of thresholds: it is the same above-mentioned interval [15, 16.5] for both *anosmia* or functional *anosmia*.

The categories of (functional) *anosmia*, *hyposmia*, *normosmia* are included in all studies. However, only two studies^9,10^ deal with the subcategories of *hyposmia* (severe, moderate, mild).

There are discrepancies in the aggregate TDI scores:

- there may be aggregated TDI scores that do not allow a diagnosis to be made (TDI = 15.25, 30.75)^11^; (TDI = 15.25, 15.5, 15.75, 30.25, 30.5, 30.75)^12,13^; (TDI = 16.5)^14,15^; (TDI = 15.25, 15.5, 15.75, 16, 34.5)^16^; (TDI = 30)^17^; (TDI = 16, 30.5, 30.75, 31)^18^; (TDI = 15.25, 15.5, 15.75, 29.25, 29.5, 29.75)^19^; (TDI = 15.25, 15.5, 15.75, 29.25, 29.5, 29.75)^20^; (TDI = 16, 31)^21^; (TDI = 30)^22^),
- categories are not exclusive, so two diagnoses can be made at the same time ((TDI = 30.5)^23^; (TDI = 16.5)^24^),
- a high *normosmia* cut-off (TDI > 34.5) compared to other studies^16^,
- TDI cut-offs for age groups in the 10th percentile of assessors^25–28^, so that the threshold and hence the diagnosis depends on the group under study,
- category thresholds that can never be TDI scores (e.g., 0.30 and 0.60 fractional points) can be specified with fractional points of 0.25, 0.50, and 0.75, respectively, allowing for equality, since only integer points can be given for identification and discrimination, and for threshold the available fractional points can only be 0.25, 0.5, 0.75^14,15,23^ (Table 1).

Based on literature research, categories of discrepancies in TDI scores have been established. Differences in the small TDI scores are significant in defining the cut-off values between *anosmia* and *hyposmia*, not only for accurate diagnosis but also for guiding the selection of appropriate therapeutic interventions.

**Table 1:** *Sniffin’ sticks* (Burghart Messtechnik GmbH, Holm, Germany) olfactory function test system summary scores (T + D + I) and diagnoses (Scores are listed in ascending order.).

Study population		anosmia	functional anosmia	hyposmia	normosmia
60 patients with idiopathic inflammatory myopathies (48 women, 12 men; 52.1 ± 14.1 years) 60 control subjects (48 women, 12 men; 50.9 ± 13.1 years)^29^	TDI cut-off scores	< 15	–	15 ≤ x ≤ 30	30 <
	Diagnosis	Patients: 2 controls: 0	–	Patients: 41 controls: 14	Patients: 17 controls: 46
193 patients: 30 phantosmia, 40 parosmia, 123 had no qualitative olfactory symptoms (116 women, 51.3 ± 2.1 years; 77 men, 49.2 ± 1.6 years)^17^	TDI cut-off scores	≤ 15	–	15 < x < 30	30 <
	Diagnosis	73	–	75	45
30 patients (14 women, 16 men; 62.3 ± 7.7 years)^30^	TDI cut-off scores	≤ 15	–	15 < x ≤ 30	30 <
	Diagnosis	22	–	8	0
280 patients with olfactory dysfunction (137 women, 143 men; 41.5 ± 15.0 years)^31^	TDI cut-off scores	≤ 15	–	15 < x < 31	31 ≤
	Diagnosis	205	–	75	0
53 patients with chronic rhinosinusitis, 36 patients with allergic rhinitis (38 women, 51 men; 39.83 ± 16.05 years) 30 healthy subjects (14 women, 16 men; 37.03 ± 13.09 years)^16^	TDI cut-off scores	≤ 15	–	16 < x < 34.5	34.5 <
	Diagnosis	Patients: 19 healthy: 0	–	Patients: 30 healthy: 0	Patients: 40 healthy: 30
40 operators (19 women, 21 men; 30–39 years were dominant) 40 non operators (20 women, 20 men; 30–39 years were dominant)^19^	TDI cut-off scores	≤ 15	–	16 ≤ x ≤ 29	30 ≤
	Diagnosis	Operators: 8 non operators: 0	–	Operators: 23 non operators: 0	Operators: 9 non operators: 40
24 patients with trans-ethmoid (14 women, 10 men; 47.0 ± 13.4 years) 24 patients with trans-sphenoethmoid (12 women, 12 men; 56.6 ± 10.8 years)^20^	TDI cut-off scores	≤ 15	–	16 ≤ x ≤ 29	30 ≤
	Diagnosis	Patients with trans-ethmoid: 8 patients with trans-sphenoethmoid: 8	–	Patients with trans-ethmoid: 13 patients with trans-sphenoethmoid: 14	Patients with trans-ethmoid: 3 patients with trans-sphenoethmoid: 2
1 patient with traumatic brain injury (woman, 40-year-old)^12^	TDI cut-off scores	≤ 15	–	16 ≤ x ≤ 30	31 ≤
	Diagnosis	1	–	0	0
1 patient with traumatic brain injury (woman, 40-year-old) 1 patient with traumatic brain injury and cervical trauma (woman, 53 year-old) 1 patient with traumatic brain injury (man, 56 year-old)^13^	TDI cut-off scores	≤ 15	–	16 ≤ x ≤ 30	31 ≤
	Diagnosis	3	–	0	0
15 patients with certain anosmia 35 healthy subjects^22^	TDI cut-off scores	≤ 15	–	< 30	30 <
	Diagnosis	15	–	0	35
130 patients with narcolepsy-cataplexy: 63 from France (37 women, 26 men; 42.9 ± 19.4 years) 129 healthy controls: 61 from France (32 women, 29 men; 33.4 ± 12.6 years), 68 from Italy (33 women, 35 men; 38.2 ± 16.1 years)^9^	TDI cut-off scores	≤ 15	–	Severe: 15 < x ≤ 20	30 <
				Moderate: 20 < x ≤ 25	
				Mild: 25 < x ≤ 30	
	Diagnosis	France patients: 0 controls: 0	–	France Patients: 0 Controls: 0	France Patients: 45 Controls: 57
				France Patients: 4 Controls: 0	
				France Patients: 14 Controls: 4	
106 patients with idiopathic REM sleep behavior disorder (19 women, 87 men; 66.6 ± 7.4 years) 28 control subjects (11 women, 17 men; 62.2 ± 3.6 years)^10^	TDI cut-off scores	≤ 15	–	Severe: 15 < x ≤ 20	30 <
				Moderate: 20 < x ≤ 25	
				Mild: 25 < x ≤ 30	
	Diagnosis	Patients: 36 controls: 0	–	Patients: 35 Controls: 0	Patients: 0 Controls: 10
				Patients: 22 Controls: 4	
				Patients: 13 Controls: 14	
20 cystic fibrosis patients with chronic rhinosinusitis without polyps with different mutations (31.5 ± 9.4 years) 20 healthy subjects (29.6 ± 6.9 years)^25^	TDI cut-off scores	< 16	–	Varies with age	
	Diagnosis	Patients: 3 healthy: 0	–	Patients: 12 Healthy: 3	Patients: 2 Healthy: 14
				Borderline (hypo-normosmic): Patients: 3 Healthy: 3	
27 patients with postinfectious olfactory loss (17 women, 10 men; 22–74 years, mean age 54.8 years)^28^	TDI cut-off scores	< 16	–	varies with age	
	Diagnosis	15	–	12	0
30 patients (21 women, 9 men; 58.1 ± 12.0 years)^32^	TDI cut-off scores	≤ 16	–	16 < x < 30.75	30.75 ≤
	Diagnosis	6	–	21	3
25 patients with loss of smell following COVID-19 classical and with olfactory training (15 women, 10 men; 38 ± 14 years) 18 patients with loss of smell following COVID-19 classical and without olfactory training group of (9 women, 9 men, 37 ± 10 years)^33^	TDI cut-off scores	≤ 16	–	16.25 ≤ x ≤ 30.5	30.75 ≤
	Diagnosis	Patients with olfactory training: 4 controls: 3	–	Patients with olfactory training: 21 controls: 15	Patients with olfactory training: 0 controls: 0
83 patients with chronic renal failure (37 women, 46 men; 19–73 years, median 52 years) 24 control subjects (14 women, 10 men; 38–75 years, median 44 years)^34^	TDI cut-off scores	–	< 15	15 ≤ x ≤ 30	30 <
	Diagnosis	–	Patients: 7	Patients: 62	Patients: 14
176 patients with olfactory dysfunction (114 women, 62 men; 57.4 ± 14.1 years)^11^	TDI cut-off scores	–	≤ 15	15.5 ≤ x ≤ 30.5	31 ≤
	Diagnosis	–	89 (53 women, 36 men)	75 (56 women, 19 men)	12 (5 women, 7 men)
27 patients with Parkinson disease (16 women, 11 men; 65.6 ± 9.7 years) 17 healthy subjects (14 women, 3 men; 61.4 ± 7.4 years)^21^	TDI cut-off scores	–	< 16	16 < x < 31	31<
	Diagnosis	–	patients: 7 healthy: 0	patients: 20 healthy: 9	patients: 0 healthy: 8
110 patients with persistent olfactory dysfunction (75 women, 35 men; 37.5 ± 11 years)^18^	TDI cut-off scores	–	< 16	16.25 ≤ x < 30.5	31<
	Diagnosis	–	33	77	0
400 patients with Parkinson’s disease (137 women, 263 men; mean age 64.3 years)^27^	TDI cut-off scores	–	< 16	varies with age	
	Diagnosis	–	180	207	13
19 patients with self-reported olfactory dysfunction (10 women, 9 men; 52.0 ± 21.7 years)^35^	TDI cut-off scores	–	≤ 16	16 < x < 30.75	30.75 ≤
	Diagnosis	–	12	7	0
203 patients with quantitative olfactory dysfunction (111 women, 92 men; 54.2 ± 16.6 years)^36^	TDI cut-off scores	–	≤ 16	16 < x < 31	31 ≤
	Diagnosis	–	87	116	0
470 patients with olfactory disorders (309 women, 161 men; 48.4 ± 16.2 years)^37^	TDI cut-off scores	–	≤ 16	16 < x < 31	31 ≤
	Diagnosis	–	129	241	100
285 patients with olfactory disorder (160 women, 125 men; 56.93 ± 14.67 years)^38^	TDI cut-off scores	–	≤ 16	16 < x < 31	31 ≤
	Diagnosis	–	128	130	27
9139 subjects (4928 women, 31.8 ± 18.9 years; 4211 men, 30.7 ± 17.7 years)^39^	TDI cut-off scores	–	≤ 16	16.25 ≤ x ≤ 30.5	30.75 ≤.
	Diagnosis	–	48	2332	6759
54 patients with olfactory loss (23 men, 39.9 ± 13.9 years; 21 women)^40^	TDI cut-off scores	–	≤ 16	16.25 ≤ x ≤ 30.5	30.75 ≤.
	Diagnosis	–	12	29	13
68 smokers (33 women, 35 men; 42.4 ± 11.6 years)^14^	TDI cut-off scores	–	< 16.5	16.5 < x < 30.3	30.3 <
	Diagnosis	–	7	54	7
28 subjects (17 women, 11 men; 44.3 ± 13 years)^15^	TDI cut-off scores	–	< 16.5	16.5 < x < 30.3	30.3 <
	Diagnosis	–	3	23	2
60 patients with olfactory disorders (25 women, 35 men; 45.7 ± 15.1 years) 60 healthy subjects (27 women, 33 men; 38.4 ± 12.0 years)^41^	TDI cut-off scores	–	< 16.5	16.5 ≤ x < 30.5	30.5 ≤
	Diagnosis	–	patients: 23	patients: 37	patients: 0
15 patients undergoing endoscopic skull base surgery sparing the olfactory tracts (11 women, 4 men; mean age 38.7 years)^26^	TDI cut-off scores	–	< 16.5	varies with age	
	Diagnosis	–	0	3	12
40 patients undergone total laryngectomy (8 women, 32 men; 65.72 ± 10.34 years)^23^	TDI cut-off scores	–	≤ 16.5	16.6 ≤ x ≤ 30.5	30.5 ≤
	Diagnosis	–	15	25	0
161 subjects (108 women, 53 men; 40 ± 18.2 years)^24^	TDI cut-off scores	–	≤ 16.5	≤ 30.5	30.5 ≤
	Diagnosis	–	4	48	109

The original recommendations for the *Sniffin’ Sticks* test empirically established a criterion of TDI ≤ 16 as the diagnosis of *anosmia*, defined as the loss of olfaction. Lötsch et al.^42^ determined the 87th percentile for the cut-off value of TDI = 16 by simulating 100,000 random completions and proposed a TDI = 17 corresponding to the 90th percentile. Their research also examined the olfactory threshold testing protocol and suggested starting the test with a medium concentration (Tstart = 8) sample instead of the original lowest concentration (Tstart = 16) sample to reduce the risk of erroneously rejecting the diagnosis of *anosmia* based on high scores arising from guessing in the olfactory threshold test.

The theoretical objectives of our research are to statistically revise the *Sniffin’ Sticks* test, in particular (1) to determine the most accurate TDI cut-off point between the diagnosis of *anosmia* and *hyposmia*, (2) to examine the number of alternatives in the identification subtest and the TDI, and (3) to ensure multiple types of equalisation of correct answers. The practical objective of our research is to ensure that, based on the results of the theoretical objectives, the protocol of the *Sniffin’ Sticks* test system can be implemented in clinical practice with minimal modification.

Objective 1 is to determine the most accurate TDI cut-off point between the diagnosis of *Sniffin’ Sticks anosmia* and *hyposmia*. This is particularly necessary because of the different values reported both in research and in clinical diagnostic practice. We further aim to refine the percentile values by simulating tests with orders of magnitude more random individuals than previously^42^. We point out that values of TDI that are excessively low should be revised to detect possible fraud. Since *anosmic* individuals cannot smell odours, their choices are random in all three subtests (T, D, I).

Our 2nd objective is to find a very low TDI threshold that could serve as a potential indicator for fraud detection. Extremely low TDI scores may warrant suspicion, as a patient might have an incentive to produce artificially poor results − for reasons such as insurance claims, patient support eligibility, or avoiding specific job requirements. The aggregate TDI score is not monotonically increasing, in the case of random selection, low values may have a low probability of occurence. The distribution plots for random choice can provide insights into the likelihood of small values.

Our 3rd objective is to reduce the likelihood of accidental hits and the risk of misdiagnosis due to the nature of the *Sniffin’ Sticks* subtests: threshold (three alternative forced choice, 3-AFC), discrimination (3-AFC), identification (4-AFC). Therefore, we aimed to determine the impact of the original *Sniffin’ Sticks* identification 4-AFC subtest on the TDI score in addition to the 5–10-AFC subtest. The practical advantage of this objective is that it does not involve any additional sensory effort, as the assessors only need to smell one odour. However, they have to select from a larger set of alternatives.

Our 4th, independent objective is to perform a statistical evaluation of the internationally culturally adapted *Sniffin’ Sticks* identification tests with emphasis on matching criteria. Specifically, we test (4a) the matching of the frequency of correct answers, (4b) the matching of the frequency of adjacent pairs of correct answers, and (4c) the matching of the correct answers over time. Our objective is to identify sample sequences that satisfy all three matching conditions. To our knowledge, no prior comparison of these tests has been conducted using to the above criteria.

## Methodology

### Anosmia threshold determination

The *Sniffin’ Sticks* test evaluates an individual’s sense of olfaction based on their achieved TDI score. The diagnosis is *anosmia*, if the TDI score is 16 or lower (see Table 1 for different cut-off values). If the individual is indeed olfactory-deprived, the responses in the different subtests must be random. According to Lötsch et al.^42^, a TDI score threshold of 16 corresponds to the 87th percentile of the distribution obtained from tests with random responses. Thus, the original test implies a false-positive rate (a type-I error rate)—the probability that a person with a loss of olfaction is incorrectly classified as not belonging to this category—of 13%. This type-I error rate is higher than those typically accepted for similar tests (1%, 5%, 10%), therefore it may be beneficial to modify the test accordingly, by considering higher quantiles (thresholds for given probabilities) of the TDI scores distribution based on random responses.

For this purpose, we implemented the protocol of the full test set in MATLAB (version 9.13.0.2049777 (R2022b) for Windows™, MathWorks, Natick, MS, USA). Using sample sizes of 100,000, 1,000,000, 10,000,000, 100,000,000 and 1,000,000,000, we examined the resulting simulated TDI distributions—i.e., the thresholds corresponding to the 90th, 95th, and higher percentiles of the points obtained—assuming completely random responses (Supplementary Information S1). Thus, in the implemented simulations, every option is chosen with the same probability for every question, i.e., the results only depend on theoretical distributions instead of data related to clinically diagnosed cases similar to Lötsch et al.^42^ This approach can only be used to determine the *anosmia* cut-off, but it is not applicable for the *normosmia* threshold, where examining clinically diagnosed cases is inevitable. The sample size allowed for highly precise determination of (possibly extremely high) quantiles (and hence the possible test thresholds).

### Response to the impact of the number of alternatives

By modifying the simulation of the *Sniffin’ Sticks* test set protocol implemented in MATLAB (version 9.13.0.2049777 (R2022b) for Windows™, MathWorks, Natick, MS, USA), we determined the distributions and various notable quantiles of the TDI scores associated with random responses by changing the number of possible alternatives in the identification subtest from 2 to 10.

The higher the number of alternatives, the lower the probability of getting the correct odour in a random response. However, this effect becomes less pronounced as the number of alternatives goes larger. Our simulation results enabled precise quantification of these effects.

### Equalisation (identification)

The *Sniffin’ Sticks* odour identification test, culturally adapted for different countries, consists of 16 questions with a four-alternative forced-choice format, which is subject to equivalence tests. Complete enumeration and filtering were performed with a custom program written in MATLAB (the same as above), using e.g., the perm command that generates all repeat permutations.

- Balanced frequency of correct answers: Determining the frequency of correct answers was based on basic data from the literature. Ideally, the positions (1st, 2nd, 3rd, 4th alternative) of correct answers are evenly distributed. For the original *Sniffin’ Sticks* test (Burghart Messtechnik GmbH, Holm, Germany), consisting of 16 odours (questions), being comprised of four target odours at the first, second, third, and forth alternatives, respectively, in the answer sheet. However, the frequencies of correct answers in some cultural adaptations are unbalanced (Section “Balanced frequency of correct answers (identification)” and Supplementary Table S2). We shall find frequency-balanced sequences among all possible sequences.
- Balance of frequencies of adjacent pairs of correct answers: Determining the frequencies of adjacent pairs of correct answers based on basic data from the literature and finding adjacent-balanced sequences among all possible sequences.
- Balance correct answers timing: Characterisation of temporal smoothing based on baseline data from the literature. Temporal evenness is defined as the number of questions in which a specific number of correct answers appear in a given number of rows with the same average value. For a test with 16 questions and 4 answer alternatives, this average is (1 + 2 + 3 …. + 16)/(4 × 4) = 8.5. Here, one factor of 4 in the denominator represents the number of answer alternatives and the other represents their frequency of occurrence. This means that the average frequency of any correct answer falls exactly between questions 8 and 9. The average deviation from perfect balance is therefore quantified by the average of the absolute distances from 8.5.
- Finding fully balanced sequences: all the three types of balancedness above can be fulfilled simultaneously. We examine all the (16!)/(4!)^4^ = 63,063,000 permutations of the multiset {1, 1, 1, 1, 2, 2, 2, 2, 3, 3, 3, 3, 4, 4, 4, 4}, and identify configurations where both the matching of adjacent response pairs and the temporal matching of the 1, 2, 3, 4 elements were simultaneously satisfied.

## Results

### Anosmia threshold determination

**Fig. 1:**
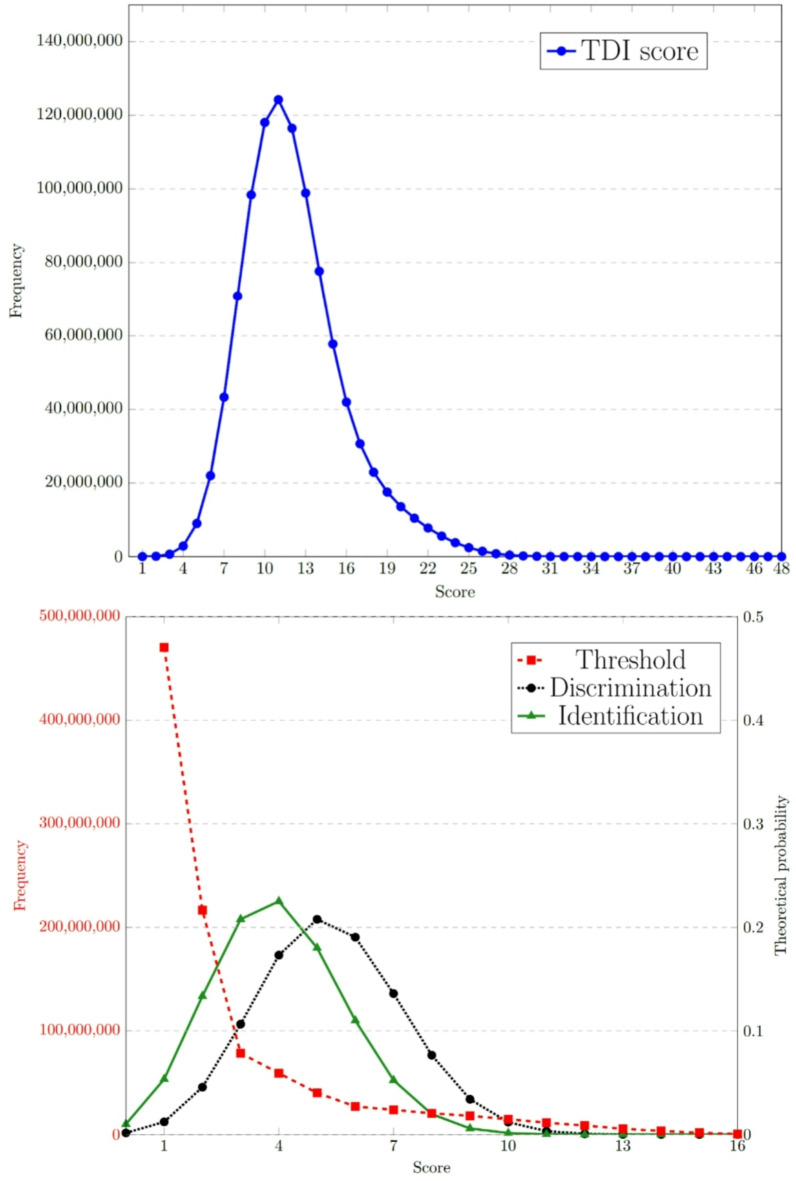
Simulated distribution of **TDI** scores from a 1 billion randomly responding sample (top), and its decomposition **T** + **D** + **I** (bottom). The simulated distribution of **Threshold** (bottom, shown in red on the left y-axis) subtest from a 1 billion sample, and theoretical distributions of **Discrimination** (bottom shown in black on the right y-axis) and **Identification** (bottom, in green on the right y-axis) subtests when the subject answers the questions randomly.

In the case of complete loss of olfactory perception (*anosmia*), the subject responds randomly in all three subtests of the *Sniffin’ Sticks* test set. The simulated distribution for the threshold (T) subtest as well as the theoretical distribution of scores for the discrimination (D ~ binomial(16,1/3)) and identification (I ~ binomial(16,1/4)) subtests, and their sum T + D + I, the total TDI score were analysed (see Fig. 1). For further details of the simulated scores, see Appendix A.

The currently used score cut-off of 16 for *anosmia* corresponds to the 89th percentile of simulated TDI scores when the subject is completely olfactory deprived and, accordingly, selects answers randomly across all subtests. This implies that the probability of diagnosing a randomly responding individual as *anosmic* by the test is 0.89. This can also be interpreted as an alpha or first-order error (the probability that a person with a loss of olfaction is incorrectly misclassified as not being *anosmic* by the test) of 11%. This is higher than the commonly accepted alpha error bounds (10%, 5%, 1%, etc.), so it may be worth testing the higher quantiles (90%, 95%, 99%, etc.). The ceilings and exact values are shown in Table 2. Although fractional values (any multiples of 0.25) are theoretically possible whole numbers are typically preferred as cut-offs, which is why the ceiling values are presented as well. One can see that, there are large differences in the possible alternative cut-off values depending on the determined quantile (e.g., the score value of 16.75 for 90% and 23 for 99%). In addition, it is important to note that TDI scores are in a sense non-monotonic, since the probability of a subject scoring extremely low in the case of a random response (i.e., completely olfactory) is very low (the expected score in this case is 11.85). However, patients may have an incentive to manipulate their results to be classified as *anosmic* (e.g. for insurance benefits). Accordingly, a very low score may not necessarily indicate anosmia (since this is very unlikely to be achieved by randomly answering) but could instead suggest uncooperative behaviour or deliberate manipulation to be detected as *anosmic*. As a form of fraud detection, it may also be worth looking at the low quantiles (0.1%, 0.5%, 1%, 2.5%, etc.). The ceilings and the exact quantiles are shown in Table 2. These quantiles show the probability of achieving a given score at most through random responses and can be used as thresholds for fraud detection. Subjects scoring below such thresholds might be required to retake the test.

**Table 2:** Ceilings and exact values of the major quantiles of the TDI score when a subject answers all questions completely randomly (uniform distribution).

Quantile	Ceiling	Exact value
0.10%	4	3.75
0.50%	5	5
1%	5	5
2.50%	6	6
5%	7	7
10%	8	8
90%	17	**16.75**
95%	19	**19**
97.50%	21	20.75
99%	23	**23**
99.50%	25	24.25
99.90%	27	26.75

The probability of an *anosmic* patient scoring less than 5 points is below 0.5%. It may therefore be worth recommending a further testing if such a low score is observed. In addition, to align with commonly accepted alpha error levels, the *anosmia* threshold should be raised, reducing the probability of mis-classifying an *anosmic* patient in another category.

In Table 2, we highlighted in bold the TDI score thresholds associated with the most frequent first-order error values: 10%: 16.75, 5%: 19, 1%: 23. As the first-order error decreases, there is a corresponding increase in the second-order error (the probability of diagnosing a *hyposmic* patient as *anosmic*), which can only be quantified by knowing the distribution of the *hyposmia* TDI score.

### Response to the number of alternatives (threshold, discrimination, Identification)

The *Sniffin’ Sticks* 3 response alternative tests (3-AFC) have a 33% chance of being hit by chance, while the 4 response alternative tests (4-AFC) have a 25% chance of being hit by chance. As the number of response alternatives increases, the probability of hitting the target by chance decreases, following a theoretical relationship described by a hyperbolic function. Thus, increasing the number of alternatives serves as an effective method to reduce the bias due to guessing.

This is straightforward for three- and four-choice alternatives in forced-choice problems, where the probability of guessing the correct answer is 1/3 and 1/4, respectively. The confidence limits are easily calculated from standard probability equations. However, determining the theoretical threshold for the olfactory threshold is more complex, since the three-choice alternative forced-choice task is embedded within a complex stepwise structure, complicating the direct application of the usual probability equations. Because of this difficulty, prior studies have reported olfactory thresholds based on observed scores of individuals who were otherwise diagnosed as *anosmic*^42^.

The identification subtest can be easily modified in a way that does not affect sensory fatigue by changing the number of possible alternatives from the current 4. As previously mentioned, the currently used score cut-off of 16 for *anosmia* ensures that a subject with complete olfactory loss (randomly responding) has an 89% probability of scoring at most this number of points. The same probabilities are presented in Table 3 for tests containing modified versions of the identification subtest. The higher the number of possible alternatives, the higher the quantile associated with the currently used score threshold.

**Table 3:** Quantiles corresponding to the currently used *anosmia* TDI score threshold of 16 for different numbers of identification alternatives, provided that the patient always answers all questions completely randomly (uniformly distributed, sample size of 1 billion).

Quantile/I-Alternative	2	3	4	5	6	7	8	9	10
16	59.56%	81.84%	88.23%	90.93%	92.38%	93.28%	93.90%	94.34%	94.68%

**Fig. 2:**
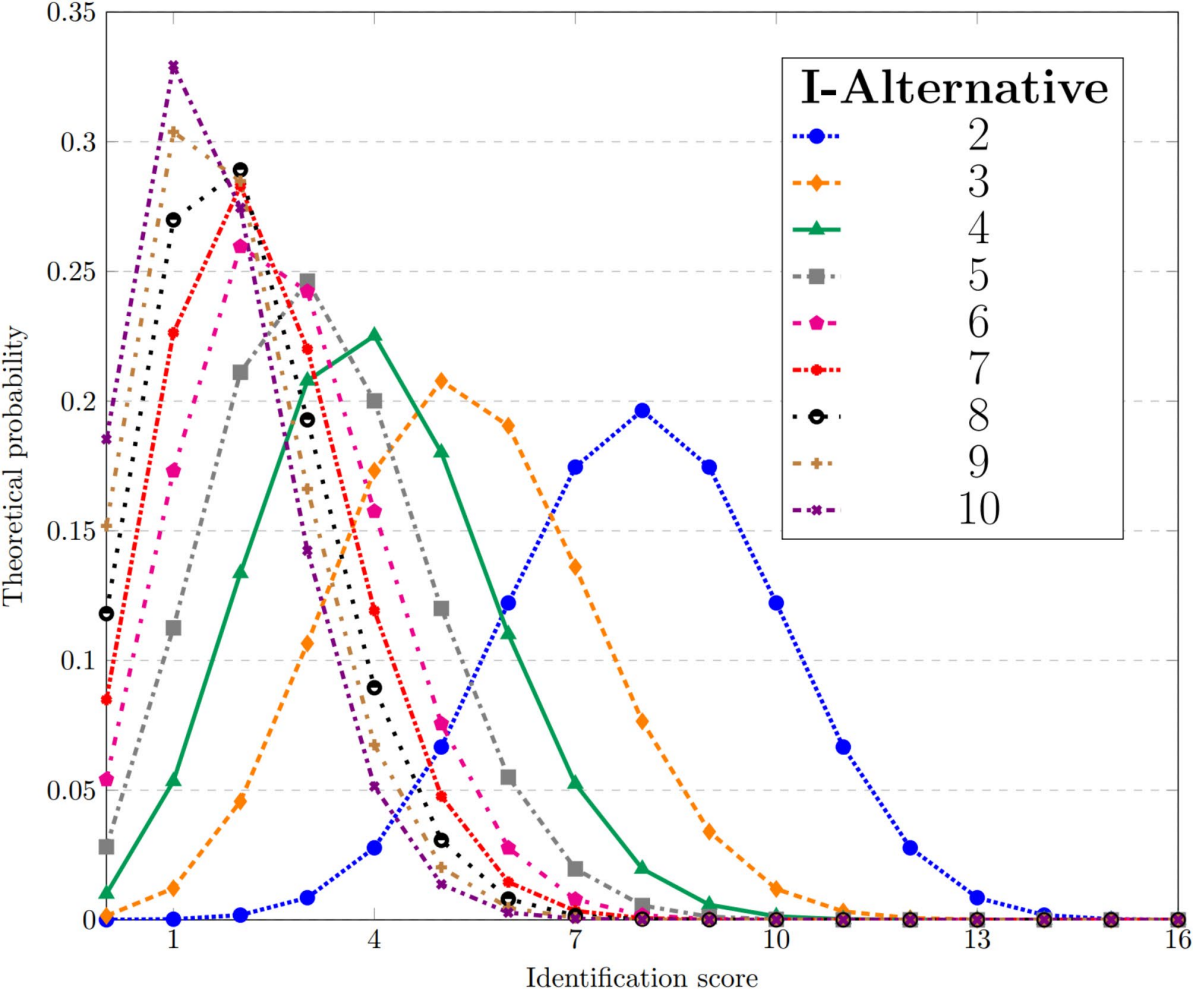
The (theoretical) distribution of the identification score for different numbers of alternatives.

**Fig. 3:**
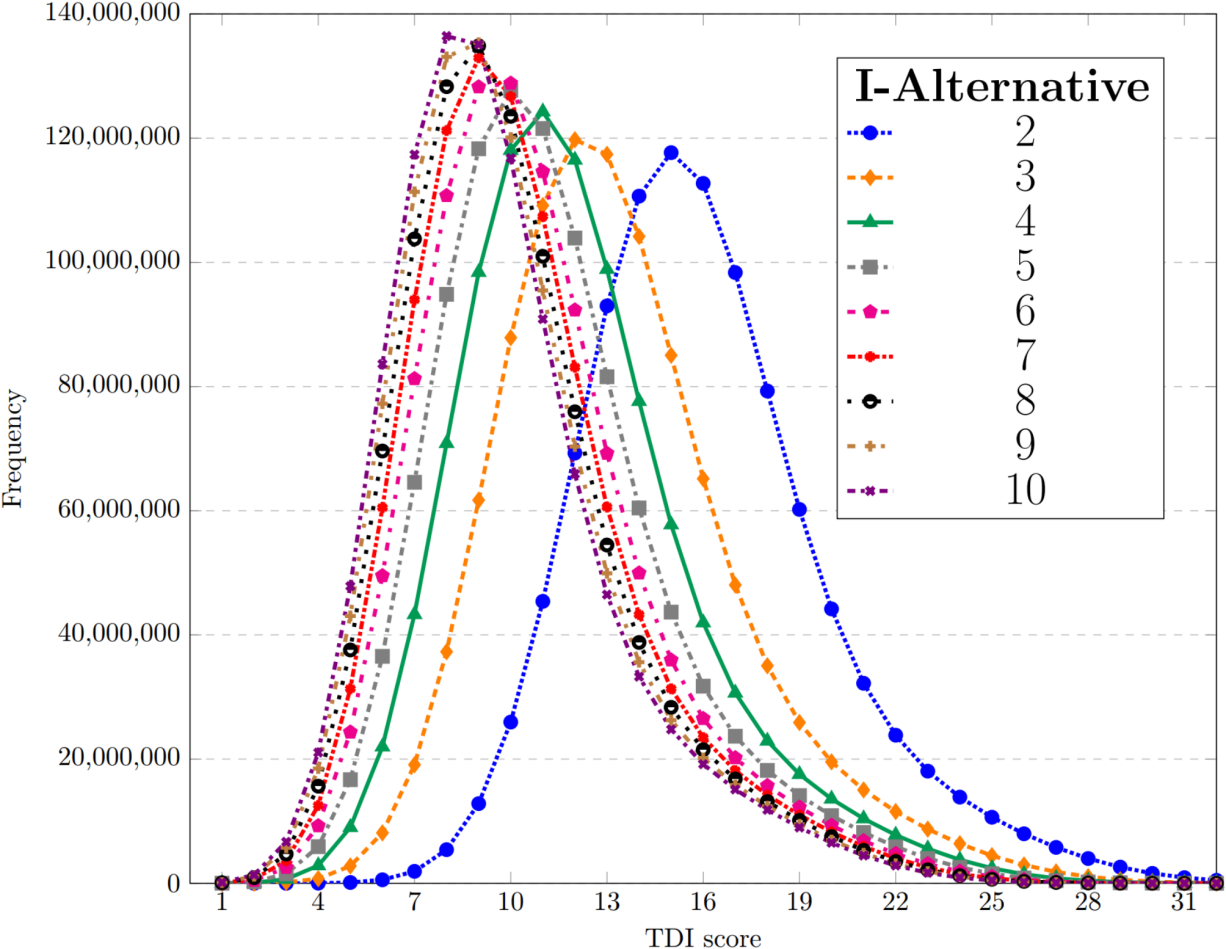
Simulated distribution of the TDI score for different numbers of alternatives in the identification test (sample size: 1 billion). Possible TDI scores range from 1 to 48, however, values above 32 are excluded from the figure due to their negligible frequency, ensuring better visibility.

The identification subtest naturally yields different point distributions for each alternative number among participants who fill in the questionnaire entirely at random. We calculated the theoretical score distributions for the identification subtest with 2–10 alternatives (Fig. 2) and simulated the distributions for the total TDI score (Fig. 3).

For the resulting distributions of TDI scores, it may be worth considering using different olfactory thresholds for diagnosing of *anosmia* and detecting fraud, as mentioned in Section “Anosmia threshold determination”, compared to those used in the original test. For example, it could be beneficial to record the value of the alpha error, i.e., the quantile associated with the olfactory threshold (and fraud detection). We simulated the exact quantile values of interest (Table 4) and the average achievable score (Table 5). It is worth highlighting that there can be a large difference between the gained quantiles that can be used as the olfactory threshold in the test, e.g., the 99th quantile is 23 in the currently used version of the test with four alternatives in the identification subtest, while it is 20.25 when there are ten alternatives in the identification subtest.

**Table 4:** The main quantiles of the TDI score for different numbers of alternatives in the identification subtest, assuming the patient answers all the questions completely randomly (uniform distribution, sample size: 1 billion).

Quantile/I-Alternative	2	3	4	5	6	7	8	9	10
0.10%	7	4.25	3.75	3	3	2.75	2.25	2	2
0.50%	8	6	5	4	4	3.25	3	3	3
1%	8.75	6	5	4.75	4	4	4	4	3.5
2.50%	9.5	7	6	5.25	5	5	4.25	4	4
5%	10.25	8	7	6	6	5.25	5	5	5
10%	11.25	9	8	7	6.5	6	6	6	6
90%	20.75	18	16.75	15.75	15.25	14.75	14.5	14.25	14
95%	23	20.25	19	18	17.5	17	16.75	16.5	16.25
97.50%	25	22.25	20.75	20	19.5	19	18.75	18.5	18.25
99%	27.25	24.5	23	22	21.5	21	20.75	20.5	20.25
99.50%	28.5	25.75	24.25	23.25	22.75	22.25	22	21.75	21.5
99.90%	31	28.25	26.75	25.75	25.25	24.75	24.25	24	23.75

**Table 5:** Average TDI scores for different numbers of identification alternatives, assuming the patient answers all the questions completely randomly (uniform distribution, sample size: 1 billion).

Quantile/I-Alternative	2	3	4	5	6	7	8	9	10
Average	15.85	13.18	11.85	11.05	10.51	10.13	9.85	9.62	9.45

Our results consistently indicate that the higher the number of possible alternatives in the identification subtest, the lower the score of the randomly selected subjects is expected to be. This results in the distribution shifting progressively toward lower scores, with all quantile values correspondingly decreasing. Additionally, it can also be observed that this effect decreases as the number of alternatives increases beyond the currently used 4. For example, the difference between alternatives 9 and 10 are small for most quantiles.

When implementing fraud detection, a viable approach may be to consider a truncated distribution, where subjects with the lowest scores are excluded when determining the upper olfactory thresholds. In such cases, a repeated test (or an alternative test) is recommended. This leads to a truncated distribution, for which we can determine the notable olfactory thresholds if we assume that each question is answered randomly by the subject. The important quantiles of the 10% truncated distribution—where the lowest 10% of scores, corresponding to subjects scoring at or below the 10% quantile (Table 4) are removed—are presented in Table 6. It can be seen, however, that the olfactory thresholds previously defined exhibit robust behaviour under this transformation, with only minor differences observed between the original olfactory thresholds (quantiles) and those calculated from the truncated distribution.

**Table 6:** Major quantiles of the 10% truncated distribution of the TDI score for different numbers of alternatives to identification, assuming the patient answers all the questions completely randomly (uniform distribution, sample size: 1 billion).

Quantile/I-Alternative	2	3	4	5	6	7	8	9	10
90%	21.25	18.5	17.25	16.25	15.5	15.25	15	14.75	14.75
95%	23.25	20.75	19.5	18.5	17.75	17.5	17.25	17	16.75
99%	27.25	24.75	23.25	22.25	21.75	21.25	21	20.75	20.5
99.90%	31	28.5	27	26	25.25	24.75	24.5	24.25	24

The quantile values can also be used to define point intervals where randomly selected patients fall with a given probability (achieved by symmetrically cutting off points with equal probability from both ends of the distribution). This result can also help in defining olfactory thresholds for the diagnosing of *anosmia* and detecting fraud (Table 7).

**Table 7:** Probability of the TDI score falling within different intervals for different numbers of identification alternatives (2–10), assuming the patient answers all questions completely randomly (uniform distribution, sample size: 1 billion).

I-Alternative									
2	3	4	5	6	7	8	9	10	Interpretation
11.25−20.75	9.00–18.00	8.00−16.75	7.00−15.75	6.50−15.25	6.00−14.75	6.00−14.50	6.00−14.25	6.00−14.00	80% the probability that the TDI score of an always guessing *anosmic* is in this range
10.25−23.00	8.00−20.25	7.00−19.00	6.00−18.00	6.00−17.50	5.25−17.00	5.00−16.75	5.00−16.50	5.00−16.25	90% the probability that the TDI score of an always guessing *anosmic* is in this range
9.50−25.00	7.00−22.25	6.00−20.75	5.25−20.00	5.00−19.50	5.00−19.00	4.25−18.75	4.00−18.50	4.00−18.25	95% the probability that the TDI score of an always guessing *anosmic* is in this range
8.75−27.25	6.00−24.50	5.00−23.00	4.75−22.00	4.00−21.50	4.00−21.00	4.00−20.75	4.00−20.50	3.50−20.25	98% the probability that the TDI score of an always guessing *anosmic* is in this range
8.00−28.50	6.00−25.75	5.00−24.25	4.00−23.25	4.00−22.75	3.25−22.25	3.00−22.00	3.00−21.75	3.00−21.50	99% the probability that the TDI score of an always guessing *anosmic* is in this range

We performed our analysis with different sample sizes, which makes it clear that this aspect has a crucial effect on the results, as in some cases a large sample size is necessary to calculate stable quantiles. For further details regarding the sample size, see Appendix B.

### Equalisation (identification)

#### Balanced frequency of correct answers (identification)

When comparing the culturally adapted *Sniffin’ Sticks* odour identification tests, we found a balanced pattern of correct responses for these adapted *Sniffin’ Sticks* tests. In contrast, the following adapted *Sniffin’ Sticks* tests were not balanced: Brazil (3-4-4-5), United Kingdom (4-5-3-4), Egypt (3-5-5-3), Republic of Korea (4-5-4-3). Among these, the Egyptian *Sniffin’ Sticks* test was the most unbalanced (Table 8).

**Table 8:** Balanced frequency of correct answer placement in 16-pen *Sniffin’ sticks* 4 response alternative tests (4-AFC) (balanced pattern placement: 4-4-4-4).

Country	Frequency of correct answers in the Sniffin’ Sticks test				Balanced frequency
	Correct answer for answer choice 1	Correct answer for answer choice 2	Correct answer for answer choice 3	Correct answer for answer choice 4	
15 countries (Germany*^43^, Democratic Republic of the Congo*^44^, Denmark*^45,46^, Greece*^47^, Iran*^48^, Malaysia*^49^, Poland*^50^, Portugal*^51^, Romania*^52^, Slovakia*^53^, Spain*^54^, Sri Lanka*^55^, Taiwan*^56^, Tanzania*^57^, Turkey*^58,59^)	4	4	4	4	yes
Brazil^60,61^	3	4	4	5	no
Egypt^62^	3	5	5	3	no
Republic of Korea^63^	4	5	4	3	no
United Kingdom^64,65^	4	5	3	4	no

*The order of the correct answers is the same as the original *Sniffin’ Sticks* (Burghart Messtechnik GmbH, Holm, Germany)^43^.

#### Equalisation of frequencies of adjacent correct answer pairs (identification)

While the frequency of correct responses is the same for the original (Burghart Messtechnik GmbH, Holm, Germany) *Sniffin’ Sticks* (16 odours, 4-4-4-4, 1, 2, 3, 4), the sequential order of correct responses varies. The theoretical goal is to have i→j pairs of answers with the same frequency (1→1, 1→2, 1→3, 1→4, 2→1, 2→2, 2→3, 2→4, 3→1, 3→2, 3→3, 3→4, 4→1, 4→2, 4→3, 4→4), if possible. The theoretical optimum is that each adjacent correct pair of answers is included once, except for one i, j pair. For the *Sniffin’ Sticks* tests used in practice, no test is uniform with respect to adjacent correct answer pairs (Table 9).

**Table 9:** Equality of frequencies of correct response pairs in 16-pen *Sniffin’ sticks* 4-AFC tests.

Country	Order of correct answers	Adjacent correct answer pairs															
		1→1	1→2	1→3	1→4	2→1	2→2	2→3	2→4	3→1	3→2	3→3	3→4	4→1	4→2	4→3	4→4
15 countries (Germany*^43^, Democratic Republic of the Congo*^44^, Denmark*^45,46^, Greece*^47^, Iran*^48^, Malaysia*^49^, Poland*^50^, Portugal*^51^, Romania*^52^, Slovakia*^53^, Spain*^54^, Sri Lanka*^55^, Taiwan*^56^, Tanzania*^57^, Turkey*^58,59^)	1 3 4 2 2 3 1 4 3 2 4 1 4 3 1 2	0	1	1	2	0	1	1	1	2	1	0	1	1	1	2	0
Brazil^60,61^	3 1 1 3 2 4 2 4 1 4 3 2 2 4 3 4	1	0	1	1	0	1	0	3	1	2	0	1	1	1	2	0
Egypt^62^	1 2 3 4 3 2 1 2 3 4 3 2 1 2 3 4	0	3	0	0	2	0	3	0	0	2	0	3	0	0	2	0
Republic of Korea^63^	1 3 2 2 2 3 1 4 3 2 4 1 4 3 1 2	0	1	1	2	0	2	1	1	2	2	0	0	1	0	2	0
United Kingdom^64,65^	1 2 4 2 2 3 1 4 3 2 4 1 4 3 1 2	0	2	0	2	0	1	1	2	2	1	0	0	1	1	2	0

*The order of the correct answers is the same as the original *Sniffin’ Sticks* (Burghart Messtechnik GmbH, Holm, Germany)^43^.

The permutation tests resulted in a total of 331,776 combinations. Among these, in addition to balanced pattern matching, the frequency of almost perfectly balanced pairs of adjacent correct answers was achieved, with only one consecutive pair always missing. However, it is useful to examine these combinations further to identify any patterns that might be recognisable for the judges. Obviously, if testers guess the order of correct answers, this could easily bias the results to the patient’s possible interests. A further research question is whether this has an impact on practical testing.

#### Time balance of correct answers (identification)

The temporal frequency of correct responses is not assured for either the original or the culturally adapted *Sniffin’ Sticks* tests (Table 10). The larger the average deviation from perfect matching (8.5), the more temporal matching is violated: Brazil (1.908), Republic of Korea (1.017), Egypt (0.800), United Kingdom (0.692), compared to the others (0.375). If the average of the sequence of correct answers at a given location (1st, 2nd, 3rd, 4th) is less than 8.5, it occurs earlier on average at that location, and if it is greater, it occurs later. With a perfectly balanced allocation over time, we can ensure that the assessors can infer the number of correct answers for each question as little as possible from their earlier (presumably correct) answers.

**Table 10:** Mean value of the Temporal occurrence of the correct answers to the 16-pen *Sniffin’ sticks* 4 response alternative tests (4-AFC) (perfectly balanced in time: correct answer in 1st position (8.5), correct answer in 2nd position (8.5), correct answer in 3rd position (8.5), correct answer in 4th position (8.5), mean deviation (0)).

Country	Order of correct answers	Average of the sequence of correct answers in a given place				Average deviation from perfect balance in time
		1st place	2nd place	3rd place	4th place	
15 countries (Germany*^43^, Democratic Republic of the Congo*^44^, Denmark*^45,46^, Greece*^47^, Iran*^48^, Malaysia*^49^, Poland*^50^, Portugal*^51^, Romania*^52^, Slovakia*^53^, Spain*^54^, Sri Lanka*^55^, Taiwan*^56^, Tanzania*^57^, Turkey*^58,59^)	1 3 4 2 2 3 1 4 3 2 4 1 4 3 1 2	8.75	8.75	7.75	8.75	0.375
Brazil^60,61^	3 1 1 3 2 4 2 4 1 4 3 2 2 4 3 4	4.667	9.25	7.75	10.8	1.908
Egypt^62^	1 2 3 4 3 2 1 2 3 4 3 2 1 2 3 4	7	8.4	8.6	10	0.800
Republic of Korea^63^	1 3 2 2 2 3 1 4 3 2 4 1 4 3 1 2	8.75	7.6	7.75	10.67	1.017
United Kingdom^64,65^	1 2 4 2 2 3 1 4 3 2 4 1 4 3 1 2	8.75	7.4	9.667	8.75	0.692

*The order of the correct answers is the same as the original *Sniffin’ Sticks* (Burghart Messtechnik GmbH, Holm, Germany)^43^.

The three equalisations (frequency, response pair, time) and the response odour alternatives of the culturally adapted *Sniffin’ Sticks* tests (Supplementary Table S2).

#### Correct answer frequency, adjacent answer pairs and Temporal matching co-completeness (identification)

**Fig. 4:**
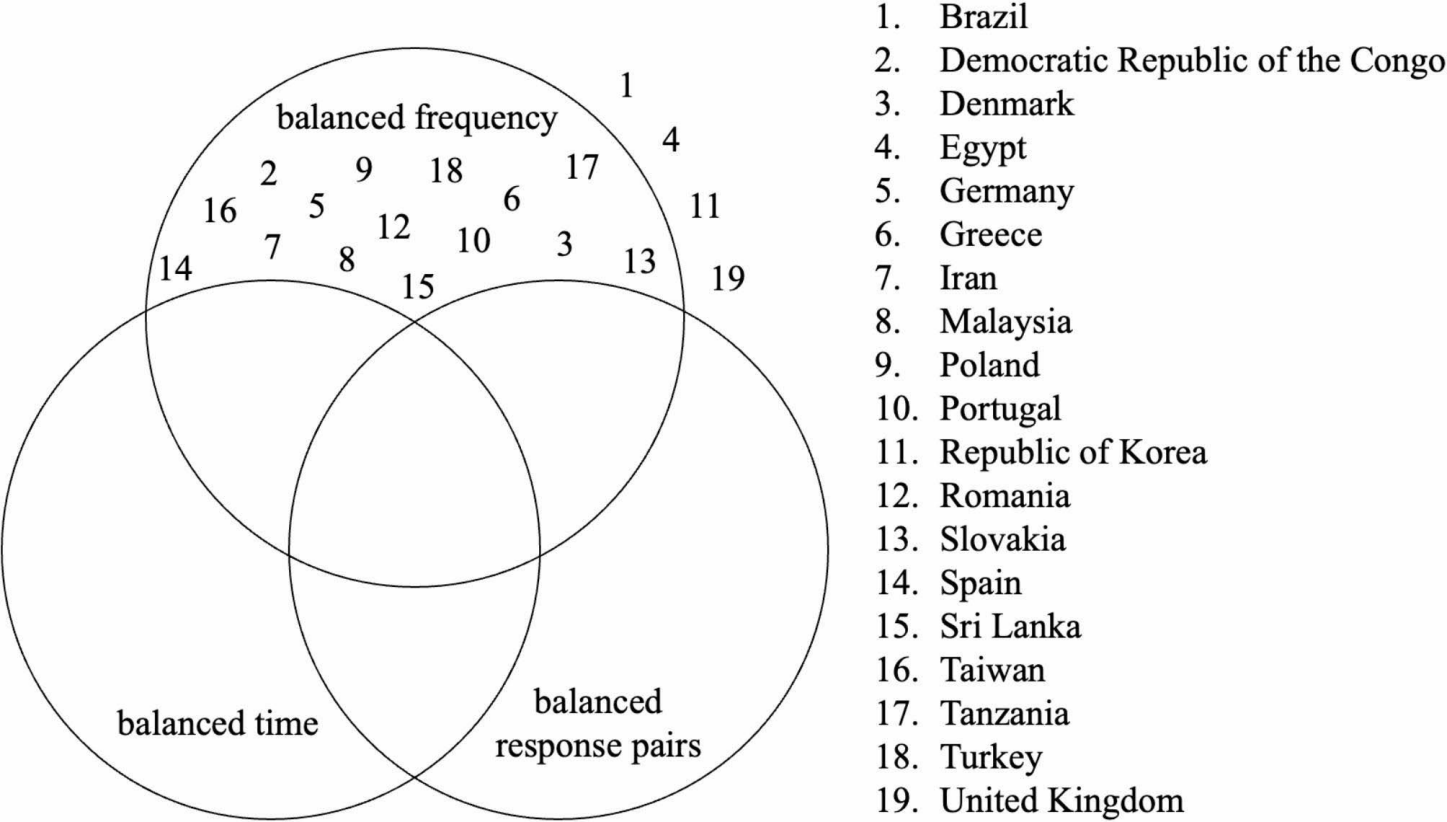
Satisfaction of the equivalences for country-adapted versions of the *Sniffin’ Sticks* identification subtest.

Based on the three criteria of balance (frequency, response pairs, time), the only adaptation of the *Sniffin’ Sticks* identification test that provided balance in some tests was the frequency of correct answers. Even this is not guaranteed in tests adapted for four countries (Brazil, Egypt, Republic of Korea, United Kingdom) (Fig. 4).

**Fig. 5:**
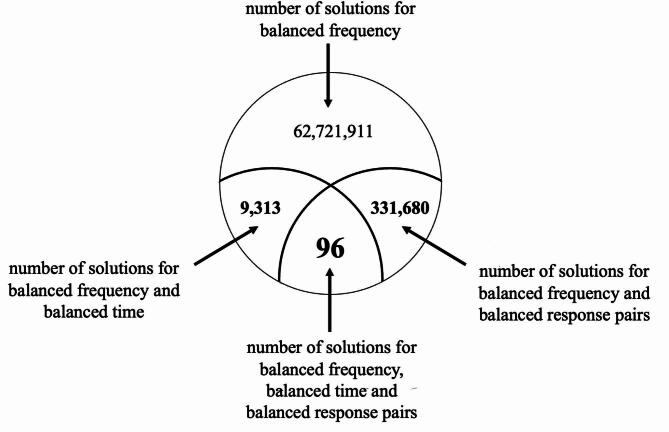
Completeness of the balanced types across all balanced frequency, balanced time and balanced response pairs sequences.

Evidently, there is a need for sequences of the *Sniffin’ Sticks* identification test that are balanced in all three criteria (frequency, response pairs, time). Among sequencies with balanced frequency, those satisfying further balance types have been enumerated. Sequences with unbalanced frequency have been further investigated. It can be observed that the number of solutions of the theoretical balancing (combinations) decreases by two orders of magnitude as follows: number of solutions for balanced frequency → number of solutions for balanced frequency and balanced response pairs → number of solutions for balanced frequency and balanced time → number of solutions for balanced frequency, balanced time and balanced response pairs (Fig. 5).

A total of 96 such sequences were found, but two further symmetries can be identified among them. On the one hand, renaming the elements {1, 2, 3, 4} according to an arbitrary permutation of their names yields a sequence which is also perfectly balanced. On the other hand, the inverse of any sequence also preserves all the balance. Based on these results, we have found 4 substantially different sequences. For practical purposes, all 96 sequences can be applied, varying from test to test as needed (Supplementary Table S3).

## Discussion

In the literature, there are inconsistent thresholds for the total TDI score and the resulting diagnoses of olfactory function on the *Sniffin’ Sticks* test system. In our study, we aimed to minimise deviations from the original protocol while defining olfactory thresholds, in particular the cut-off point between *anosmia* and *hyposmia*, as precisely as possible. Our calculations were performed on sample sizes of 100,000, 1,000,000, 10,000,000, 100,000,000 and 1,000,000,000. The results indicate increasing the sample size up to 100,000,000 is advisable, as no difference in scores for the different quantiles were observed beyond this threshold. Simulations with samples of 100,000,000 and smaller can only be compared if this limitation is taken into account. We have shown the effect of the sample size of the simulations (Appendix B), which is particularly significant at the edges of the distributions. The sample size of 100,000,000 instead of the 100,000 previously used^42^ is sufficiently large. Our analyses cover all values of the number of response alternatives (2–10) at the different significance levels tested (0.10%, 0.50%, 1%, 2.50%, 5%, 10%, 90%, 95%, 97.50%, 99%, 99.50%, 99.90%).

The standard *Sniffin’ Sticks* test olfactory thresholds for *anosmia*-*hyposmia* were determined to be 16.75 points (90%), 19 points (95%) and 23 points (99%). These results differ from previously published and commonly used olfactory thresholds. The proposed olfactory threshold modifications can be directly used in clinical diagnostic practice, even by re-evaluating previously performed tests. In clinical practice, a score at the 90% confidence level is typically used to define the cut-off points. Therefore, we recommend that a score at the 90% confidence level be used: *anosmia* ≤ 16.75 points, *hyposmia* ≥ 17 points. Thus, the diagnosis of patients close to the cut-off may differ from the most commonly used 16 point olfactory threshold, since, for example, a 16.5 point olfactory threshold would result in a diagnosis of *hyposmia* with the previous olfactory threshold, whereas our proposed olfactory threshold would result in *anosmia*. Further research is needed to clarify if the number of patients for whom the diagnosis differs between the current and proposed olfactory thresholds can be more accurately determined by knowing the empirical distribution of TDI scores.

In our research, we proposed balance measures for the frequency, timing, and balance of adjacent answer pairs of correct responses. We defined sequences that were simultaneously optimal or near optimal in these balance aspects. Eliminating unbalances ensures that patients are unable to infer the correct answers. These sequences can be directly applied in practice without modifying the other elements of the original protocol.

In *Sniffin’ Sticks* olfactory threshold subtest 3-AFC (probability of guessing the correct answer is 1/3), there are no odour-containing pens next to an odour concentration, so it may be appropriate to test these with 4-AFC (1/4), 5-AFC (1/5), 6-AFC (1/6), 7-AFC (1/7), 8-AFC (1/8), 9-AFC (1/9), 10-AFC (1/10), as well as specified tetrad (1/6), specified 2-out-of-5 (1/10), specified hexagon (1/20). In the *Sniffin’ Sticks* discrimination subtest 3-AFC (probability of guessing the correct answer is 1/3), all pens contain an odour. Therefore, test methods involving too many odours are not an option. The following methods may be appropriate: 4-AFC (1/4), 5-AFC (1/5), specified tetrad (1/6), specified 2-out-of-5 (1/10). In *Sniffin’ Sticks* odour identification subtest 4-AFC (probability of guessing the correct answer is 1/4), only a pen containing an odour needs to be smelled, and the odour is identified from the described response alternatives. The probability of guessing the correct answer cannot be reduced in the same way by using methods where several odours have to be selected. For this reason, testing them can only be feasible by increasing the number of response alternatives: 5-AFC (1/5), 6-AFC (1/6), 7-AFC (1/7), 8-AFC (1/8), 9-AFC (1/9), 10-AFC (1/10), etc. ^67^. In conclusion, the probability of guessing the correct answer is reduced when the number of response alternatives is increased. Although the sensory effort remains the same (one smelling is required), the increased number alternatives might increase the cognitive efforts and testing time. Any change in the protocol should take into account several factors, including sensory and mental fatigue, the need to maintain motivation and concentration, the practical difficulties of clinical practice, and the potentially increased testing duration. This could increase the effect of individual cognitive function shifting the focus away from purely evaluating olfactory performance.

In odour identification tasks, if among the alternative choices, some odours can be excluded, i.e., they are non-real alternatives, then the probability of guessing the correct answer is increased (according to the hyperbolic function). The presence of non-real alternatives biases the measurement results upwards, so that patients will give better than real odour identification results. Gudziol and Hummel^68^ showed that the use of non-real response alternatives improved odour identification results in *hyposmic* individuals but did not cause a change in *anosmic* individuals. The aim may be to make the questions for a particular odour equally difficult. A future research question is to express and measure the difficulty and distance between odours and to develop possible odour distance metrics.

As every scientific work, this study also has some potential limitations. First, we only use simulated data, thus, we have no information about how this directly relates to the scores of clinically diagnosed cases. As we mentioned earlier, our approach, based on random responses, can only be used for the determination of the cut-off regarding *anosmia*. In the case of the cut-offs between (i) *anosmia* and functional *anosmia*, (ii) functional *anosmia* and *hyposmia*, (iii) *hyposmia* and *normosmia*, it is necessary to analyse sample data from clinically diagnosed cases. Regarding fraud detection, it could also be interesting to check the extreme differences in the results of the different subtests. For other versions of the test, where the number of pens used differs from 16, the results cannot be applied directly, only our proposed approach can be adapted. Lastly, there is no information about the magnitude of the bias of the unbalanced tests presented in this study. However, it is known that this bias can be eliminated by the solutions proposed here.

## Conclusion

Different *Sniffin’ Sticks* total TDI score cut-offs and different ranges have been reported in the literature, leading to inconsistencies and potential misdiagnosis. To address this issue, a comprehensive statistical evaluation of the probability of guessing the correct answer was conducted, resulting in several important findings.

1. By implementation and simulation of the test protocol, we were able to determine the most accurate thresholds (quantiles) of the aggregate TDI score between the diagnosis of *anosmia* and *hyposmia*, which are higher than the currently used cut-off scores. It was pointed out that scores in *anosmic* patients do not behave monotonically: with very low scores being rare. As a result, lower thresholds for fraud detection were defined, below which it is recommended to repeat the test (or employ alternative tests).
2. The number of alternatives in the identification subtest can be easily modified without affecting sensory fatigue. Increasing the number of response alternatives decreases the probability of guessing the correct answer, following a hyperbolic function. We determined the effect of the *Sniffin’ Sticks* identification subtest on the 2–10-AFC TDI and the quantile of the resulting distributions that can be used as *anosmic* and fraud detection thresholds. It is observed that the more alternatives to choose from, the lower points the random choosers typically score, and the quantiles of the point distributions decrease. This effect, however, decreases when a high number of alternatives are already initially tested.
3. The original *Sniffin’ Sticks* identification subtest (Burghart Messtechnik GmbH, Holm, Germany), consisting of 16 pens, was adapted to populations in several countries for cultural reasons. The different adaptations are based on the German *Sniffin’ Sticks* identification test, but several elements of the test were changed during the adaptation—odours, answer choices, synonyms, order of odours, order of answer choices, order of correct answers, total number of odours to be identified—which changed the balance of correct answers in several aspects. In this context according to our results: (1) the frequency of correct responses was equalised in the assignments corresponding to the original German test, (2) the equalisation of the frequency of adjacent correct response pairs was not met in any test, and (3) the temporal occurrence of correct responses was not met in any test. We determined the order of all correct answers that simultaneously satisfied all three matching conditions.

## Electronic supplementary material

Below is the link to the electronic supplementary material.

Supplementary Material 1

Supplementary Material 2

Supplementary Material 3

Supplementary Material 4

Supplementary Material 5

## Data Availability

Data is provided within the manuscript or supplementary information files.
