# Supplementary material for: Statistical overview of the Sniffin’ sticks olfactory test from the perspectives of anosmia and hyposmia
Source: Sci Rep. 2025 Mar 15;15:8984. doi: 10.1038/s41598-025-93380-z (PMC11910658; doi:10.1038/s41598-025-93380-z)
Supplement: Supplementary file 1 — Supplementary Material 1 [file 41598_2025_93380_MOESM1_ESM.pdf]

% Supplementary 1 - Matlab code

% MatLAB implementation and simulation of the Sniffin' Sticks threshold, discrimination, identification test on different sample sizes (100,000, 1,000,000, 10,000,000, 100,000,000, 1,000,000,000).

```
clear all;

%Determining the sample size
samplesize=1000000000;

%Initialization of the results, the number in the names means the number of
%alternatives in the Identification subtest
%result2=zeros(samplesize,1);
%result3=zeros(samplesize,1);
%result4=zeros(samplesize,1);
%result5=zeros(samplesize,1);
%result6=zeros(samplesize,1);
%result7=zeros(samplesize,1);
%result8=zeros(samplesize,1);
%result9=zeros(samplesize,1);
result10=zeros(samplesize,1);

%For cicle for the number of alternatives in the Identification subtest
%Because of the potential large size of the results, it can be better to run this one by one
for identi = 10:10
for indexi = 1:samplesize

%The parameters of the discrimination subtest
hitD = 1/3;             %The possibility that we hit the right answer in the Discrimination subtest
Dpoint = 0;             %The number of scored points in the Discrimination subtest
experimentnumberD = 16; %How many experiments we run in the Discrimination subtest
```

```
%The parameters of the Identification subtest
hitI = 1/identi;       %The possibility that we hit the right answer in the Identification subtest
Ipoint = 0;            %The number of scored points in the Identification subtest
experimentnumberI = 16; %How many experiments we run in the Identification subtest
%%
%Simulation of the Threshold subtest
hit = 1/3;             %The possibility that we hit the right answer
level = 16;            %The level of concentration (the higher the level, the smaller the concentration)
round = 1;             %In which round we are
roundpoint = 0;        %What is the score of the round
points = zeros(1,7);   %The scores of all rounds (initialization)

%First round
if round == 1

  %While there was no roundpoint, and we have not reached level 0
  while roundpoint == 0 && level > 0

   if rand() <= hit

      %If we hit it twice in a row, there is a roundpoint
      if rand() <= hit
         roundpoint = level;
      else %If not, the the concentration parameter is lowered by 2 levels
         level = level-2;
      end;
   else
      level = level-2;
   end;
```

```
  end;


  %We save the score, if it would be 0 points, then it will be 1, and we modify the level of concentration
   if level == 0
       points(round) = 1;
       roundpoint = 1;
   else
   points(round) = roundpoint;
   end;
end;



%2nd round
round = 2;


%We are running 7 rounds
while round <= 7


%In the even rounds we examine higher levels of concentration parameters
if mod(round,2) == 0


   if level < 16
   level = level + 1;
   end;


  %While the roundpoint is the same as in the previous round, and we have
  %not reached level 16 yet
  while roundpoint == points(round-1) && level <16


   if rand() <= hit
```

```
      %If we hit it twice in a row, then we increase the level of the concentration
      %parameter
      if rand() <= hit
        level = level + 1;
      else %If not, then there is a roundpoint
        %We handle the case, when the subject did not hit the right
        %answer even for the lowest level of the concentration parameter
       if level>1
          roundpoint = level;
       else
          roundpoint = 0;
       end;
      end;
   else %We handle the case, when the subject did not hit the right
       %answer even for the lowest level of the concentration parameter
      if level>1
           roundpoint = level;
       else
          roundpoint = 0;
       end;
   end;

  end;

%We handle the case, when the subject hit the right
%answer even for the highest level of the concentration parameter
if level == 16
   roundpoint = 16;
end;
```

```
 %We handle the case, when the subject did not hit the right
 %answers even for the lowest level of the concentration parameter
 if level == 1
     roundpoint = 1;
 end;

 %We save the score
    points(round) = roundpoint;

 %Increase the number of rounds by 1
 round = round + 1;
end;


%When we are in an odd round
if mod(round,2) == 1

    %Decreasing the parameter of the concentration level by 1
    if level > 1
    level = level - 1;
    end;

%While the roundpoint is the same as in the previous round, and the concentration level is at least 1
%we decrease the parameter of the concentration level
while roundpoint == points(round-1) && level >=1
if rand() <= hit

        %If we hit the right answer twice, then there is a roundpoint
        if rand() <= hit
            roundpoint = level;
        else %If not, then we further decrease the concentration level
```

```
            level = level-1;
        end;
    else
        level = level-1;
    end;
end;

%Saving the score, and handling the case of level 0
if level == 0
     points(round) = 1;
     roundpoint = 1;
else
     points(round) = roundpoint;
end;

round = round +1;
end;
end;



Tpoint=(points(4) + points(5) + points(6) + points(7))/4;

%%

%Simulation of the discrimination subtest

for indexj = 1:experimentnumberD
if rand() <= hitD
    Dpoint = Dpoint+1;
end;
end;
```

```
%%

%Simulation of the identification subtest

for indexj = 1:experimentnumberI
if rand() <= hitI
   Ipoint = Ipoint+1;
end;
end;


%%

score(indexi) = Tpoint + Dpoint + Ipoint;

end;

if identi==2
result2(:,1)=score;
end;

if identi==3
result3(:,1)=score;
end;

if identi==4
result4(:,1)=score;
end;
```

```
if identi==5
result5(:,1)=score;
end;


if identi==6
result6(:,1)=score;
end;

if identi==7
result7(:,1)=score;
end;

if identi==8
result8(:,1)=score;
end;

if identi==9
result9(:,1)=score;
end;

if identi==10
result10(:,1)=score;
end;

end;

%Saving the results
%This line should be modified according to the currently saved case.
save('TDI_1000_000_000_10.mat','result10','-v7.3')
%save('TDI_1000_000_000_9.mat','result9','-v7.3')
```

```
%save('TDI_1000_000_000_8.mat','result8','-v7.3')
%save('TDI_1000_000_000_7.mat','result7','-v7.3')
%save('TDI_1000_000_000_6.mat','result6','-v7.3')
%save('TDI_1000_000_000_5.mat','result5','-v7.3')
%save('TDI_1000_000_000_4.mat','result4','-v7.3')
%save('TDI_1000_000_000_3.mat','result3','-v7.3')
%save('TDI_1000_000_000_2.mat','result2','-v7.3')
```
