# Supplementary material for: Statistical overview of the Sniffin’ sticks olfactory test from the perspectives of anosmia and hyposmia
Source: Sci Rep. 2025 Mar 15;15:8984. doi: 10.1038/s41598-025-93380-z (PMC11910658; doi:10.1038/s41598-025-93380-z)
Supplement: Supplementary file 2 — Supplementary Material 2 [file 41598_2025_93380_MOESM2_ESM.docx]

**Appendix A**

The possible values for the threshold subtest also contain fractional values (any multiples of 0.25 between 1 and 16), which is not highlighted in the main text **(Figure 1)** for better visibility. Here we show all the possible values for the threshold subtest **(Figure A1)**.


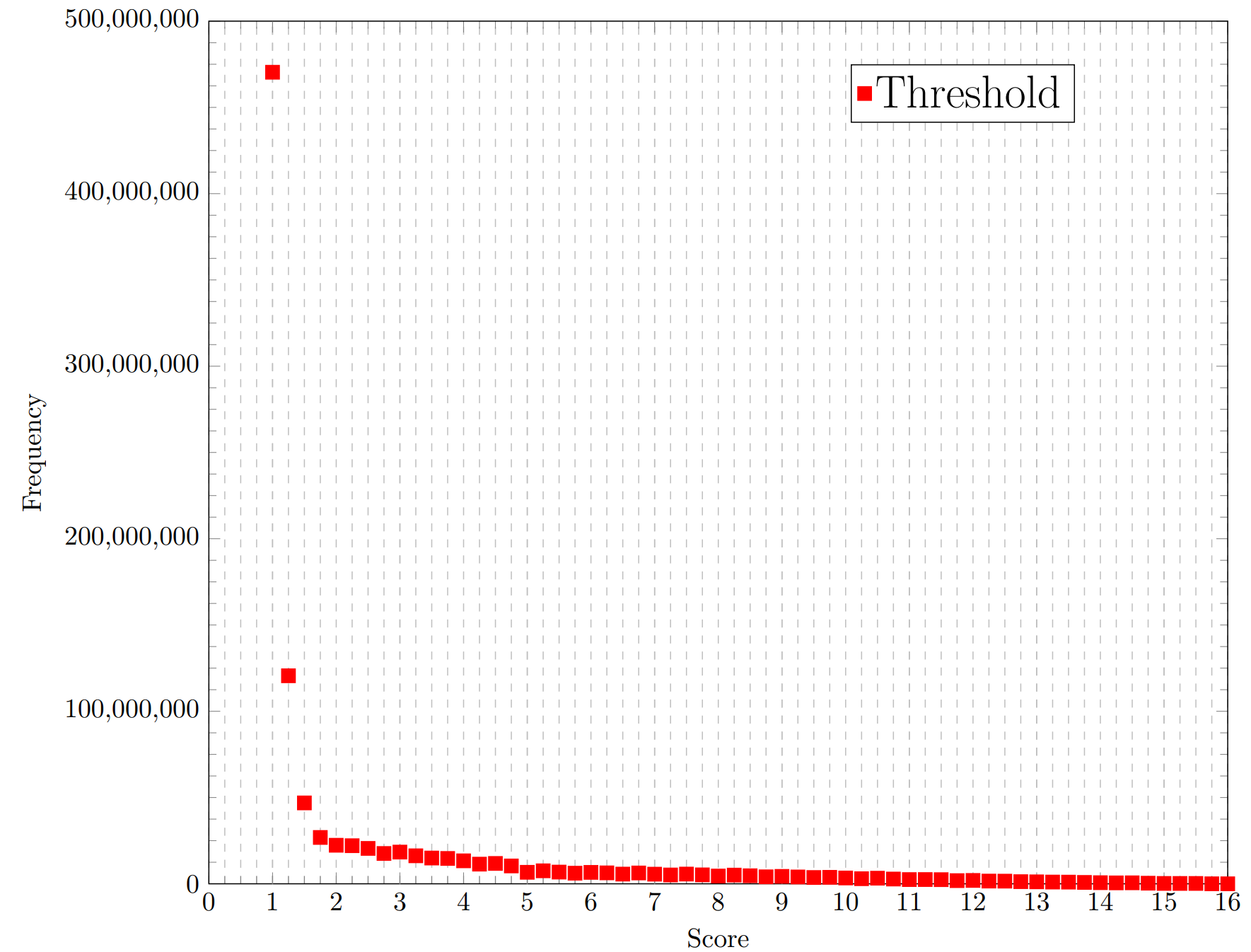


**Figure A1.** Generated threshold scores based on a sample of 1,000,000,000 observations with all possible values.

This also implies that the TDI scores can take fractional values as well (any multiples of 0.25 between 1 and 48). However, these fractional parts are directly related to the threshold subtest, as the identification and discrimination subtests can only result in whole numbers. As the 1 value is significantly more likely than any other score in the case of the threshold subtest, the TDI scores are more likely to take integer values as well **(Figure A2)**.


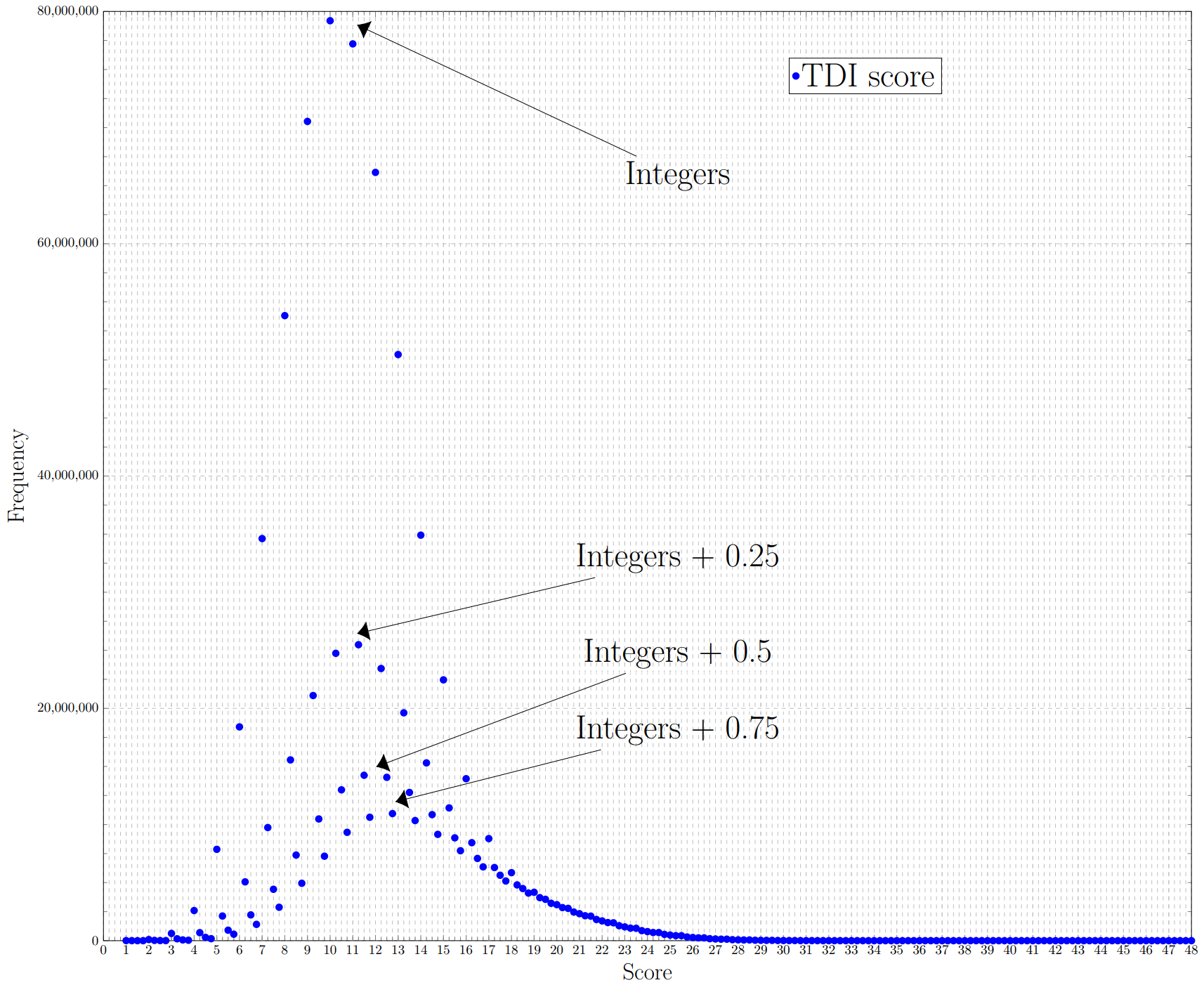


**Figure A2.** Generated TDI scores based on a sample of 1 billion observations with all the possible values.

In the main text **(Figure 1)** the frequencies of the fractional values were always assigned to their ceiling values.
