# Supplementary material for: Statistical overview of the Sniffin’ sticks olfactory test from the perspectives of anosmia and hyposmia
Source: Sci Rep. 2025 Mar 15;15:8984. doi: 10.1038/s41598-025-93380-z (PMC11910658; doi:10.1038/s41598-025-93380-z)
Supplement: Supplementary file 3 — Supplementary Material 3 [file 41598_2025_93380_MOESM3_ESM.docx]

**Appendix B**

Our calculations were also performed on the following sample sizes: 100,000, 1,000,000, 10,000,000, 100,000,000 and 1,000,000,000. The values in **Table 4** also depend on the sample sizes, but no differences were observed between the results from 100,000,000 and 1,000,000,000 samples. This indicates that increasing the sample size beyond 100,000,000 does not yield different results. For a specific quantile and number of alternatives, the effect of sample size on the TDI threshold is shown by dividing the 1,000,000,000 sample into subsamples of 100,000 observations. The results demonstrate that thresholds derived from a sample size of 100,000 are not yet stable, and two results from such samples cannot be directly compared. This also explains why previous results based on a sample size of 100,000 differ significantly from ours, as shown by the distribution in **Figure 4**.

To illustrate, the 1,000,000,000 sample dataset was divided into 10,000 subsamples of 100,000. The distribution of the latter’s TDI thresholds at the 2-alternatives identification test and 1% quantile (where we observed the largest difference between a sample size of 100,000 and 1,000,000) is shown in **Figure B1**.


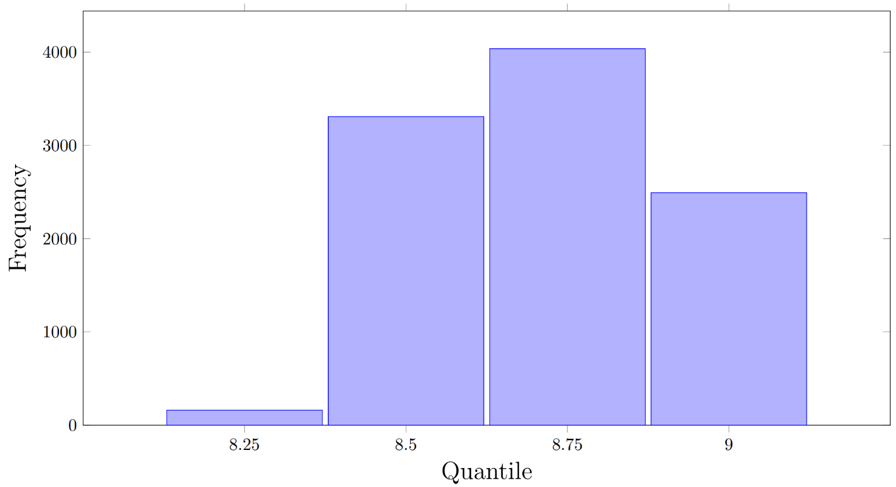


**Figure B1.** Histogram of the 1% quantile of the 1,000,000,000 observations, divided into 10,000 samples of 100,000, for the case of 2 alternatives in the identification (2-alternatives).

Our findings regarding sample size are also relevant for the alternative number 4 used in the standard *Sniffin’ Sticks* test identification subtest: the threshold for a quantile of 97.5% stabilizes at a sample size of 100,000,000. MATLAB implementation and simulation of the *Sniffin’ Sticks* threshold, discrimination, identification test on different sample sizes (100,000, 1,000,000, 10,000,000, 100,000,000, 1,000,000,000) are provided in Supplementary Information S1.
